# Neuroendocrine carcinoma of the minor papilla with pancreas divisum: a case report and review of the literature

**DOI:** 10.1186/s13256-023-03828-x

**Published:** 2023-03-28

**Authors:** Kenta Saito, Yoichi Matsuo, Yuki Denda, Keisuke Nonoyama, Hiromichi Murase, Tomokatsu Kato, Yuichi Hayashi, Hiroyuki Imafuji, Mamoru Morimoto, Ryo Ogawa, Hiroki Takahashi, Shuji Takiguchi

**Affiliations:** Department of Gastroenterological Surgery, Nagoya City University Graduate School of Medical Sciences, 1 Kawasumi, Mizuho-cho, Mizuho-ku, Nagoya, Aichi 4678601 Japan

**Keywords:** Neuroendocrine carcinoma, Neuroendocrine tumor, Minor papilla, Pancreas divisum, Pancreatitis

## Abstract

**Background:**

Neuroendocrine tumors of the minor papilla are very rare, and only 20 cases have been reported in the literature. Neuroendocrine carcinoma of the minor papilla with pancreas divisum has not been reported previously, making this the first reported case. Neuroendocrine tumors of the minor papilla have been reported in association with pancreas divisum in about 50% of cases reported in the literature. We herein present our case of neuroendocrine carcinoma of the minor papilla with pancreas divisum in a 75-year-old male with a systematic literature review of the previous 20 reports of neuroendocrine tumors of the minor papilla.

**Case presentation:**

A 75-year-old Asian man was referred to our hospital for evaluation of dilation of the main pancreatic duct noted on abdominal ultrasonography. Magnetic resonance cholangiopancreatography and endoscopic retrograde cholangiopancreatography showed a dilated dorsal pancreatic duct, which was not connected to the ventral pancreatic duct; however, it opened to the minor papilla, indicating pancreas divisum. The common bile duct had no communication with the pancreatic main duct and opened to the ampulla of Vater. A contrast-enhanced computed tomography scan showed a 12-mm hypervascular mass near the ampulla of Vater. Endoscopic ultrasonography showed a defined hypoechoic mass in the minor papilla with no invasion. The biopsies performed at the previous hospital found adenocarcinoma. The patient underwent a subtotal stomach-preserving pancreaticoduodenectomy. The pathological diagnosis was neuroendocrine carcinoma. At the 15-year follow-up visit, the patient was doing well with no evidence of tumor recurrence.

**Conclusion:**

In our case, because the tumor was discovered during a medical check-up relatively early in the course of disease, the patient was doing well at the 15-year follow-up visit, with no evidence of tumor recurrence. Diagnosing a tumor of the minor papilla is very difficult because of the relatively small size and submucosal location. Carcinoids and endocrine cell micronests in the minor papilla occur more frequently than generally thought. It is very important to include neuroendocrine tumors of the minor papilla in the differential diagnosis of patients with recurrent pancreatitis or pancreatitis of unknown cause, especially for patients with pancreas divisum.

## Background

Tumors of the minor papilla are very rare. Neuroendocrine tumors (NETs), which include somatostatinomas and carcinoid tumors, are the majority of tumors of the minor papilla [1–9]. NETs are usually located in the appendix, ileum, and rectum [10, 11]. NETs of the minor papilla are extremely rare, and about 20 cases have been reported in the literature. However, neuroendocrine carcinoma (NEC) of the minor papilla has not been reported previously.

Pancreas divisum is the most common congenital variant of the pancreas; it occurs when the embryological ventral and dorsal parts of the pancreas fail to fuse [11, 12]. Thus, pancreatic drainage occurs mainly through the dorsal pancreatic duct and the minor papilla in these patients. Previous reports have described the association of tumors of the minor papilla with pancreas divisum, but only 9 cases of NETs of the minor papilla with pancreas divisum have been reported in the literature [1–9].

We report a very rare case of NEC of the minor papilla with pancreas divisum and summarize the clinical features of NETs of the minor papilla reported in the medical literature.

## Case presentation

**Fig. 1:**
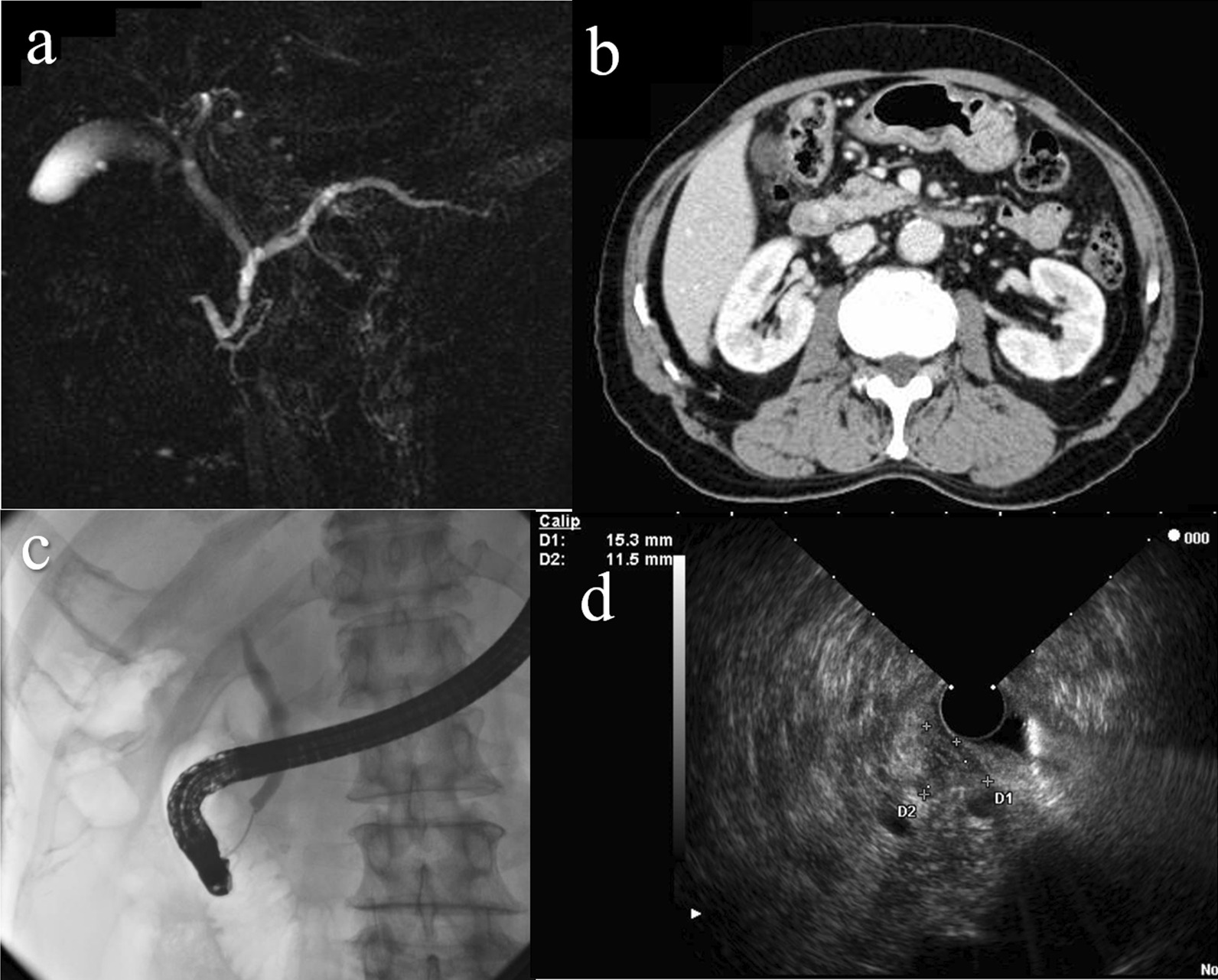
**a** Magnetic resonance cholangiopancreatography showing a dilated dorsal pancreatic duct that was not connected with the ventral pancreatic duct and opened to the minor papilla. The common bile duct had no communication with the pancreatic main duct and opened to the ampulla of Vater. **b** Contrast-enhanced computed tomography scan showing a 12-mm hypervascular mass near the ampulla of Vater and the dilated main pancreatic duct. **c** Injection of contrast through the minor papilla revealing the dilated dorsal pancreatic duct. **d** Endoscopic ultrasonography showing a defined hypoechoic mass in the minor papilla with no invasion

**Fig. 2:**
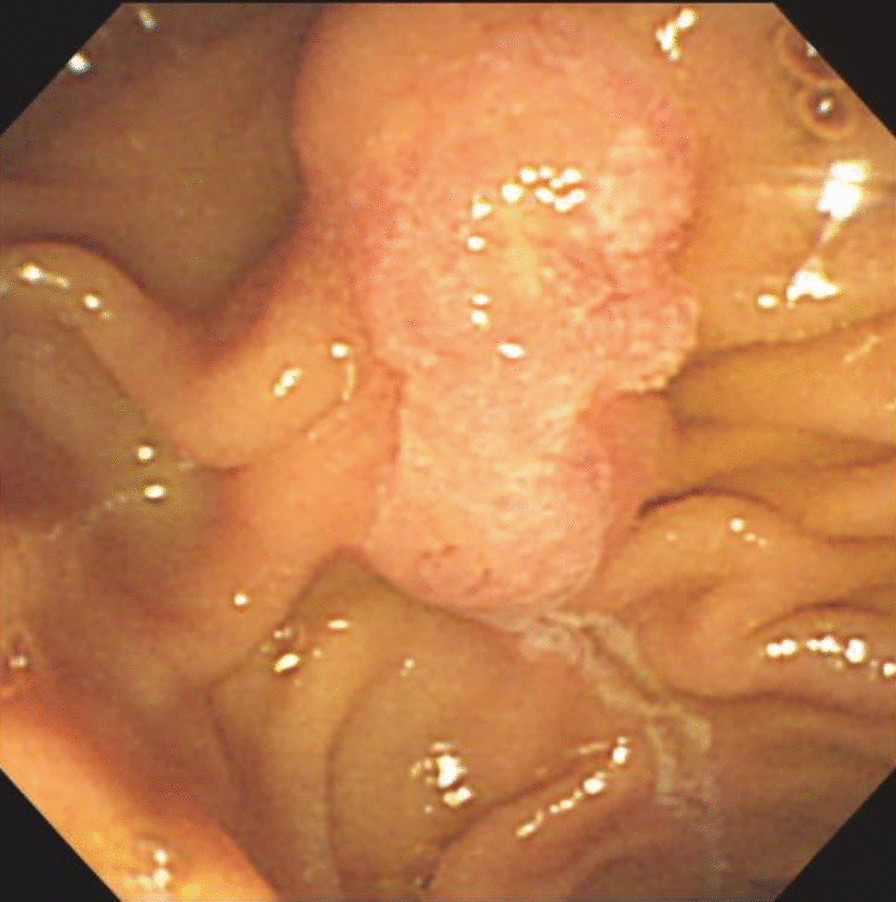
Endoscopic retrograde cholangiopancreatography showing an irregular mucosal surface in the minor papilla

A 75-year-old Asian man was referred to our hospital for evaluation of dilation of the main pancreatic duct noted on abdominal ultrasonography. There were no subjective symptoms at all. The patient’s clinical history included only hypertension and there was no relevant family history. His regular medication was only carnaculin for hypertension. The patient had smoked for 30 years (1 pack per day) until the age of 55 but had no history of alcohol. On admission, there were no physical and neurological findings. Blood pressure was 128/80 and pulse was 60 beats per minute. Laboratory data were within normal limits. Carbohydrate antigen (CA) 19-9 and carcinoembryonic antigen (CEA) were 1.0 U/mL and 1.6 ng/mL, respectively. Magnetic resonance cholangiopancreatography (MRCP) showed a dilated dorsal pancreatic duct, which was not connected with the ventral pancreatic duct, and opened to the minor papilla, indicating pancreas divisum (Fig. 1a). The common bile duct had no communication with the pancreatic main duct and opened to the ampulla of Vater. Contrast-enhanced computed tomography (CT) scan showed a 12-mm hypervascular mass near the ampulla of Vater and the dilated main pancreatic duct (Fig. 1b). Endoscopic retrograde cholangiopancreatography (ERCP) showed an irregular mucosal surface in the minor papilla (Fig. 2). Injection of contrast through the ampulla of Vater revealed the common bile duct with no dilation and inferior branches of the pancreatic duct. Injection of contrast through the minor papilla revealed the dilated main pancreatic duct (Fig. 1c). Endoscopic ultrasonography (EUS) showed a defined hypoechoic mass in the minor papilla, with no invasion and flow from the dilated pancreatic duct into the minor papilla (Fig. 1d). The biopsies performed at the previous hospital showed adenocarcinoma.

Because of the diagnosis of adenocarcinoma of the minor papilla with pancreas divisum, the patient underwent a subtotal stomach-preserving pancreaticoduodenectomy. About 10 days after the operation, leakage of the choledochojejunostomy occurred. The patient improved with conservative treatment, and he was discharged. At 15 years of follow-up, the patient was doing well with no evidence of tumor recurrence.

### Pathological findings

**Fig. 3:**
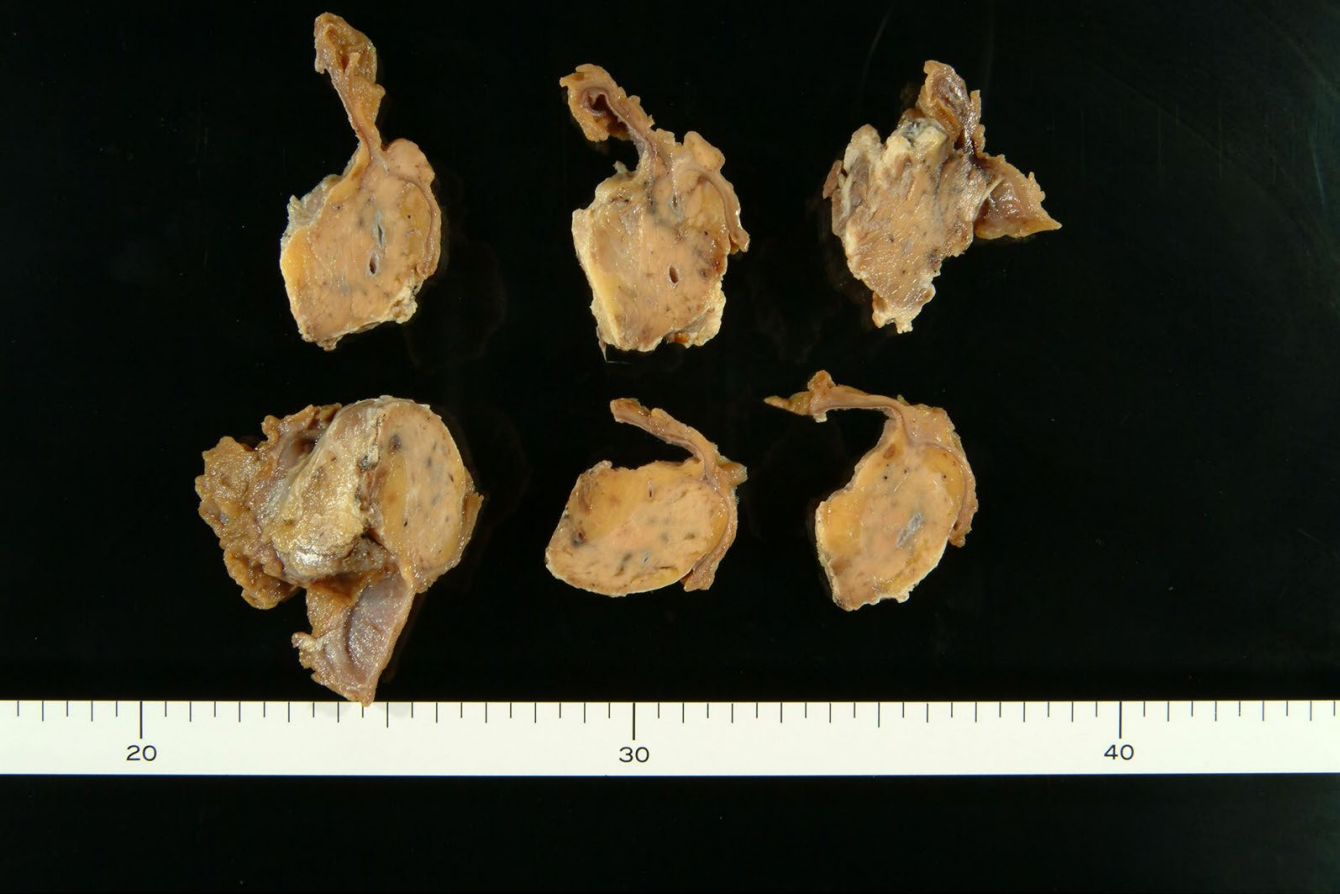
The largest tumor diameter was 25 × 15 mm in the cross-sectioned specimen, and the tumor was located in the minor papilla

**Fig. 4:**
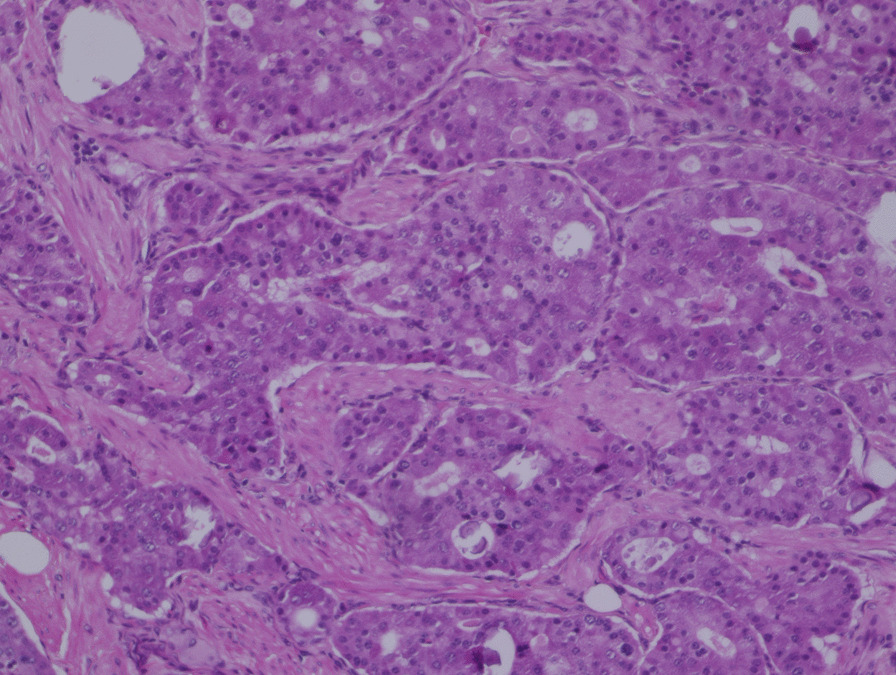
Histologically, there was infiltration of trabecular tumor with central round nuclei, coarse-clustered chromatin, abundant granules in the cytoplasm, and a low-to-intermediate N/C ratio (×200)

**Fig. 5:**
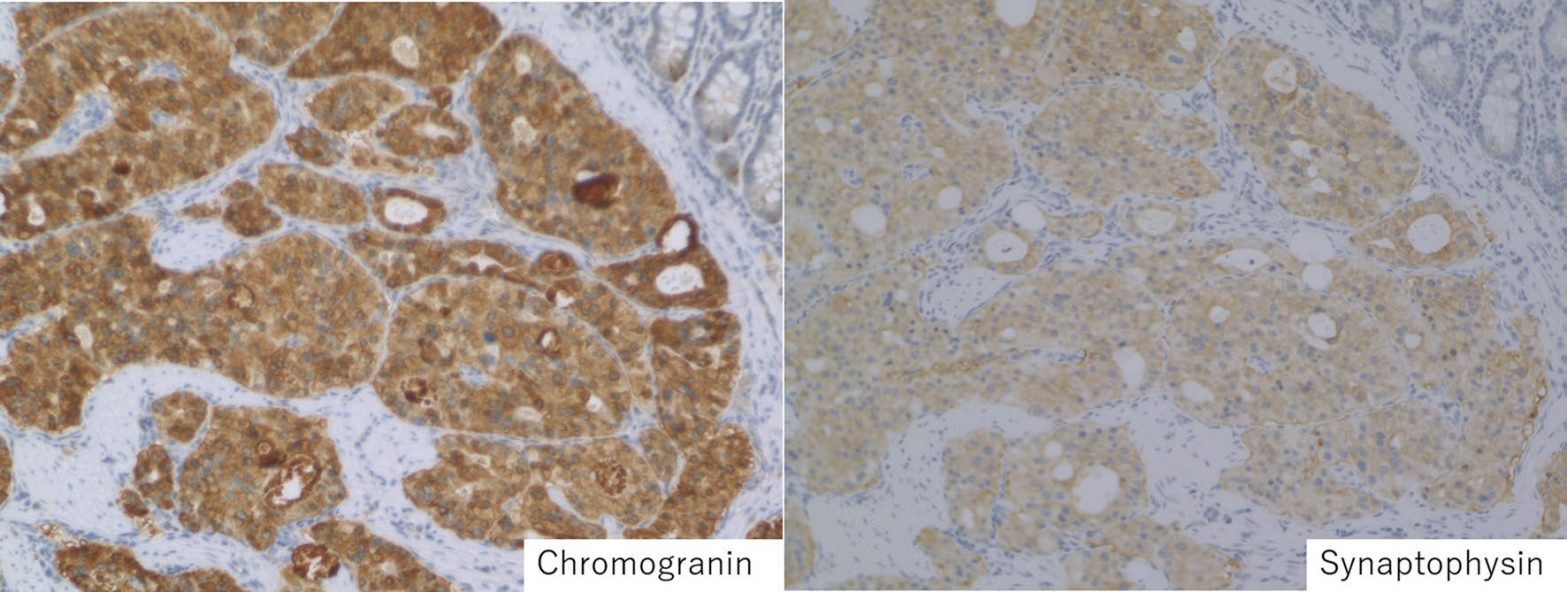
Immunohistochemical staining showing that chromogranin and synaptophysin were positive

On gross examination, the tumor was 25 × 15 mm and was located in the minor papilla (Fig. 3). Histologically, there was infiltration of trabecular tumor with abundant granules in the cytoplasm and a low-to-intermediate nuclear-cytoplasmic (N/C) ratio (Fig. 4). The tumor showed invasion of the duodenal mucosa and submucosal layer. A submuscular lymph node was also involved. Immunohistochemical staining showed that chromogranin, synaptophysin, and CD56 were positive; however, CD10, Alpha fetoprotein, glucagon, gastrin, somatostatin, and insulin were negative (Fig. 5). The pathological diagnosis was neuroendocrine carcinoma.

## Discussion and conclusions

This is a unique case of NEC of the minor papilla with pancreas divisum. NETs located in the minor papilla are very rare, and a thorough review of the literature revealed only 20 cases. The clinical features of these patients are summarized in Table 1. However, NEC of the minor papilla has not been reported previously; therefore, our patient is the first reported case. Furthermore, there are no reports of cases of NEC complicated with pancreas divisum.

**Table 1:** Summary of the cases of NETs of the minor papilla reported in the world literature

No.	Year	Author	Age (years)	Sex	Chief complaint	Size (mm)	Metastasis	Treatment	Pancreas divisum
1	1985	Malone	46	M	Epigastric pain	7.5	No	Local resection	No
2	1987	Stammer	56	M	Weight loss, jaundice	3	No	Pancreatoduodenectomy	Yes
3	1988	Lowes	50	F	Abdominal pain, weight loss	12	Lymph nodes	Pancreatoduodenectomy	Yes
4	1989	Heidt	50	M	Asymptomatic	5	Lymph nodes	Pancreatoduodenectomy	No
5	2001	Borobia	46	F	Diarrhea	No data	No	Local resection	No
6	2003	Singh	35	F	Abdominal pain	10	No	Local resection	Yes
7	2004	Outtas	45	F	Nodular panniculitis	6	No	Pancreatoduodenectomy	Yes
8	2005	Wang	50	M	Melena	9	No	Local resection	No
9	2006	Waisberg	57	F	Epigastric pain, diarrhea, weight loss	27	No	Pancreatoduodenectomy	Yes
10	2007	Itoi	65	M	Asymptomatic	12	No	Endoscopic papillectomy	No
11	2007	Bettini	47	M	Melena	12	Lymph nodes/liver	Pancreatoduodenectomy	No
12	2010	Kim	56	F	Epigastric pain	12	Lymph nodes	Pancreatoduodenectomy	Yes
13	2010	Maruyama	52	M	Epigastric pain	13	Lymph nodes	Pancreatoduodenectomy	No
14	2011	Perez	80	F	Hematemesis	12	No	Endoscopic papillectomy	No
15	2013	Fukami	71	M	Asymptomatic	12	Lymph nodes	Pancreatoduodenectomy	No
16	2014	Barresi	61	F	No data	25	No	No treatment	Yes
17	2014	Aktas	77	F	Abdominal pain, jaundice	12	No	Pancreatoduodenectomy	No
18	2015	Bhandari	50	F	Abdominal pain	17	No	Local resection	Yes
19	2016	Letelier	60	F	Epigastric pain	20	No	Endoscopic papillectomy	Yes
20	2016	Virgilio	59	M	Asymptomatic	25	Lymph nodes	Pancreatoduodenectomy	No
21	2017	Present case	75	M	Asymptomatic	25	No	Pancreatoduodenectomy	Yes

NETs: Neuroendocrine tumors, M: Male, F: Female

In our analysis of the 20 previously reported cases of NETs in the minor papilla, plus our case of NEC, we found the following. The mean age was 56.6 (range 35–80) years, and patients included 10 men and 11 women. However, in the gender distribution of patients who also had pancreas divisum, a marked female dominance was observed: 8 women, 2 men. The mean largest diameter of the tumors was 13.8 (range 3–27) mm. The most common clinical presentation was abdominal pain in nine cases (43%), and five cases were asymptomatic (24%). Lymph node metastases were diagnosed in seven cases (33%) and liver metastasis in one case (5%). Surgical procedures were pancreatoduodenectomy in 12 cases (57%), local resection in 5 cases (24%), and endoscopic papillectomy in 3 cases (14%). Previous reports indicated that tumors of the duodenal papilla have high rates of metastasis [13], so Whipple operation is the most appropriate treatment for ampullary tumors [14]. In our case, the cancer diagnosis occurred relatively early because the patient had a medical check-up. The patient underwent pancreaticoduodenectomy, and at 15 years of follow-up, the patient was doing well with no evidence of tumor recurrence.

The relationship between NETs of the minor papilla and pancreas divisum has been described previously. The smaller caliber of the accessory duct and the minor papilla may induce pancreatitis in patients with pancreas divisum [3, 15–17]. There is a possibility that the inflammation of the pancreas with pancreas divisum influences the formation of endocrine cell micronests (ECMs), which are thought to be precursor lesions of NETs of the minor papilla. Regardless of the presence of pancreas divisum, carcinoids and ECMs in the minor papilla occur more frequently than generally thought. In a study of single surgical specimens and autopsies, the incidence of carcinoids and neoplastic ECMs of the minor papilla could reach 10%. Furthermore, carcinoids in the minor papilla are twice as common as carcinoids of the major papilla, and neoplastic ECMs of the minor papilla are found five times as often [18]. The number of reported cases of NETs of the major papilla exceeds those of the minor papilla. This discrepancy may be explained by the fact that due to ampullary obstruction, tumors of the major papilla are more likely to cause symptoms, such as jaundice or abdominal pain, whereas patients with minor papillary tumors usually remain asymptomatic because there is no biliary or pancreatic obstruction.

It is very important to make a diagnosis before performing surgery because it may change the course of treatment. However, NETs of the minor papilla are very difficult to diagnose because these lesions tend to be small and usually located in the submucosal area. NETs of the minor papilla are rarely accompanied by endocrine manifestations [13]. Hence, deep biopsy samples should be obtained in such cases. A high index of suspicion must be maintained for such lesions in the appropriate setting, such as the young patient without common risk factors for pancreatitis [3]. NETs of the minor papilla should be included in the differential diagnosis of patients with recurrent pancreatitis or pancreatitis of unknown cause, especially for patients with pancreas divisum; furthermore, patients with pancreas divisum require careful follow-up to monitor for the existence or formation of NETs of the minor papilla. We think that patients with pancreaticoduodenectomy may need closer monitoring to see how often NETs develop and if any screening protocol can be established.

## Data Availability

The datasets used and analyzed during the current study are available from the corresponding author on reasonable request.

## Abbreviations

NETs: Neuroendocrine tumors
NEC: Neuroendocrine carcinoma
MRCP: Magnetic resonance cholangiopancreatography
CT: Contrast-enhanced computed tomography
ERCP: Endoscopic retrograde cholangiopancreatography
EUS: Endoscopic ultrasonography
ECMs: Endocrine cell micronests
